# Supplementary material for: PA6 and Halloysite Nanotubes Composites with Improved Hydrothermal Ageing Resistance: Role of Filler Physicochemical Properties, Functionalization and Dispersion Technique
Source: Polymers (Basel). 2020 Jan 15;12(1):211. doi: 10.3390/polym12010211 (PMC7023541; doi:10.3390/polym12010211)
Supplement: Supplementary file 1 [file polymers-12-00211-s001.pdf]

polymers

MDPI

Supplementary

PA6 and Halloysite Nanotubes Composites with Improved Hydrothermal Ageing Resistance: Role of Filler Physicochemical Properties, Functionalization and Dispersion Technique

Valentina Sabatini [1,2,*], Tommaso Taroni [1,2], Riccardo Rampazzo [1,2,3], Marco Bompieri [1], Daniela Maggioni [1], Daniela Meroni [1,2], Marco Aldo Ortenzi [1,2,3] and Silvia Ardizzone [1,2,3]

[1] Dipartimento di Chimica, Università degli Studi di Milano, Via Golgi 19, 20133 Milano, Italy; tommaso.taroni@unimi.it (T.T.); riccardo.rampa@gmail.com (R.R.); marco.bompieri@studenti.unimi.it (M.B.); daniela.maggioni@unimi.it (D.M.); daniela.meroni@unimi.it (D.Me.);marco.ortenzi@unimi.it (M.A.O.); silvia.ardizzone@unimi.it (S.A.)

[2] Consorzio Interuniversitario per la Scienza e Tecnologia dei Materiali (INSTM), Via Giusti 9, 50121 Firenze, Italy;

[3] CRC Materiali Polimerici "LaMPo", Dipartimento di Chimica, Università degli Studi di Milano, Via Golgi 19, 20133 Milano, Italy.

* Correspondence: valentina.sabatini@unimi.it; Tel.: +39-02-503-14115

Supporting Information

Table S1. Injection moulding parameters for PA6 nanocomposites specimen preparation.

Parameter	Operative data
Sample temperature	255–275 °C
Mould temperature	80 °C
Injection volume	44 cm^3
Injection pressure	1500 bar
Injection rate	40 cm^3/s
Holding pressure	400 bar
Holding time	30 s
Cooling time	30 s

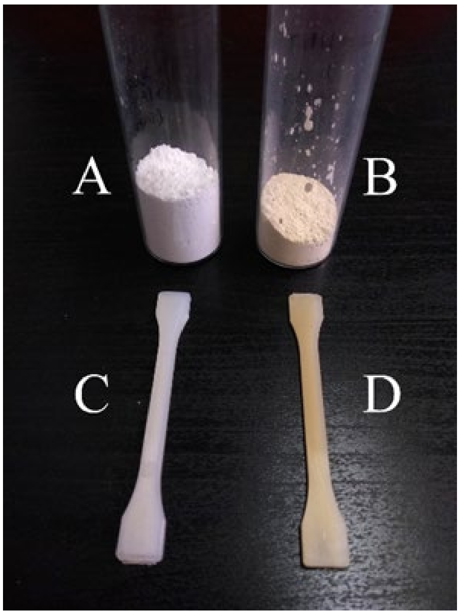

Figure S1. Visual comparison of HNT_S (**A**) and HNT_H (**B**) and their respective composites prepared via injection moulding: $PA6_{melt}$_HNT_S_4 (**C**) and $PA6_{melt}$_HNT_H_4 (**D**).

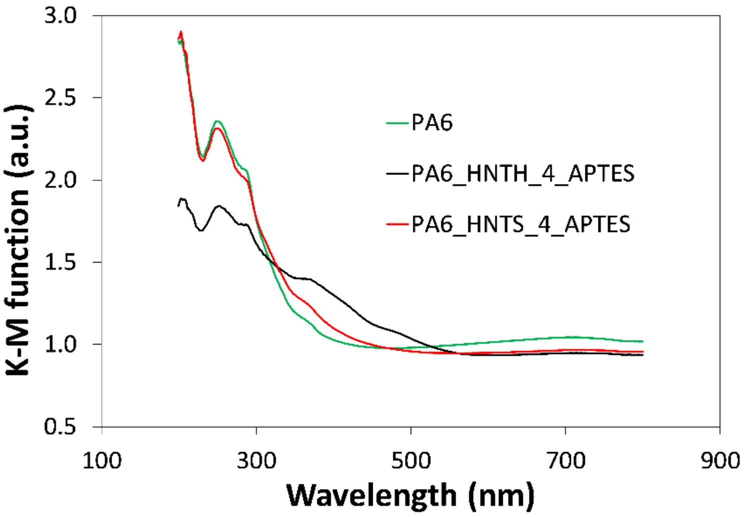

Figure S2. DRS spectra of neat, PA6_HNT_H_4_APTES and PA6_HNT_S_4_APTES nanocomposites prepared via melt blending.

Table S2. Crystallization enthalpy (ΔH_c) and heat of fusion (ΔH_f) of unaged and aged PA6_HNT nanocomposites collected during cooling and second heating thermal steps.

**Sample**	**cooling**	**2nd heating**
	**ΔH_c (J/g)**	**ΔH_f (J/g)**
PA6$_{in\ situ}$	73.3	−60.4
PA6$_{in\ situ}$_HNT$_H$_1	63.1	−57.0
PA6$_{in\ situ}$_HNT$_S$_1	57.3	−53.6
PA6$_{in\ situ}$_HNT$_H$_4	61.0	−56.5
PA6$_{in\ situ}$_HNT$_S$_4	58.5	−53.7
PA6$_{in\ situ}$_HNT$_H$_1_APTES	54.1	−51.5
PA6$_{in\ situ}$_HNT$_S$_1_APTES	69.2	−43.8
PA6$_{in\ situ}$_HNT$_H$_4_APTES	52.9	−33.6
PA6$_{in\ situ}$_HNT$_S$_4_APTES	58.6	−35.7
PA6$_{melt}$	71.7	−59.9
PA6$_{melt}$_HNT$_H$_1	65.1	−48.3
PA6$_{melt}$_HNT$_S$_1	63.5	−58.3
PA6$_{melt}$_HNT$_H$_4	62.2	−41.1
PA6$_{melt}$_HNT$_S$_4	69.7	−52.4
PA6$_{melt}$_HNT$_H$_1_APTES	64.9	−58.5
PA6$_{melt}$_HNT$_S$_1_APTES	59.0	−63.1
PA6$_{melt}$_HNT$_H$_4_APTES	69.5	−52.0
PA6$_{melt}$_HNT$_S$_4_APTES	65.1	−46.4

Table S3. Weight of the PA6_HNT nanocomposites collected during the hydrothermal ageing test at different times.

**Sample**	**weight (g)**	**weight (g)**	**weight (g)**	**weight (g)**
	**0 days**	**25 days**	**50 days**	**70 days**
PA6$_{in\ situ}$	14.39136	15.78833	14.53639	13.57776
PA6$_{in\ situ}$_HNT$_H$_1	14.73355	14.75058	14.69237	13.93750
PA6$_{in\ situ}$_HNT$_S$_1	14.26413	14.46217	14.40418	13.46581
PA6$_{in\ situ}$_HNT$_H$_4	13.51417	13.80918	13.68247	13.21360
PA6$_{in\ situ}$_HNT$_S$_4	13.62848	13.982774	13.65311	12.87875
PA6$_{in\ situ}$_HNT$_H$_1_APTES	14.78595	15.00673	14.75758	14.03173
PA6$_{in\ situ}$_HNT$_S$_1_APTES	12.53389	13.63417	12.62229	10.64601
PA6$_{in\ situ}$_HNT$_H$_4_APTES	11.87400	11.90282	11.83065	11.82596
PA6$_{in\ situ}$_HNT$_S$_4_APTES	12.81676	12.84718	12.78034	11.88061
PA6$_{melt}$	12.51653	13.8525	12.98502	12.49943
PA6$_{melt}$_HNT$_H$_1	12.27060	12.56998	12.35126	12.15381
PA6$_{melt}$_HNT$_S$_1	12.83466	14.1918	12.82144	12.76107
PA6$_{melt}$_HNT$_H$_4	12.00417	13.10212	12.95485	11.52131
PA6$_{melt}$_HNT$_S$_4	12.20384	13.95077	13.00279	12.87224
PA6$_{melt}$_HNT$_H$_1_APTES	11.78777	12.84055	12.49464	11.80357
PA6$_{melt}$_HNT$_S$_1_APTES	12.11331	13.20204	13.06264	12.81039
PA6$_{melt}$_HNT$_H$_4_APTES	13.47101	15.57738	15.4168	14.87602
PA6$_{melt}$_HNT$_S$_4_APTES	12.42536	13.96671	13.2583	12.08642

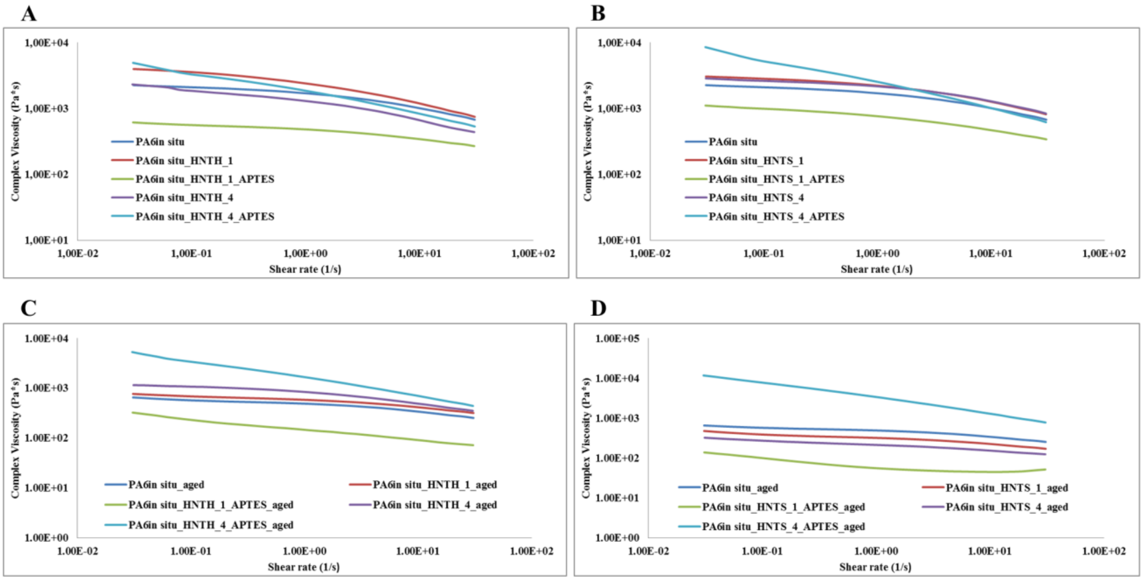

Figure S3. Rheological curves of **A**) PA6$_{in\ situ}$_HNT$_H$, **B**) PA6$_{in\ situ}$_HNT$_S$, **C**) PA6$_{in\ situ}$_HNT$_H$_aged and **D**) PA6$_{in\ situ}$_HNT$_S$_aged samples.

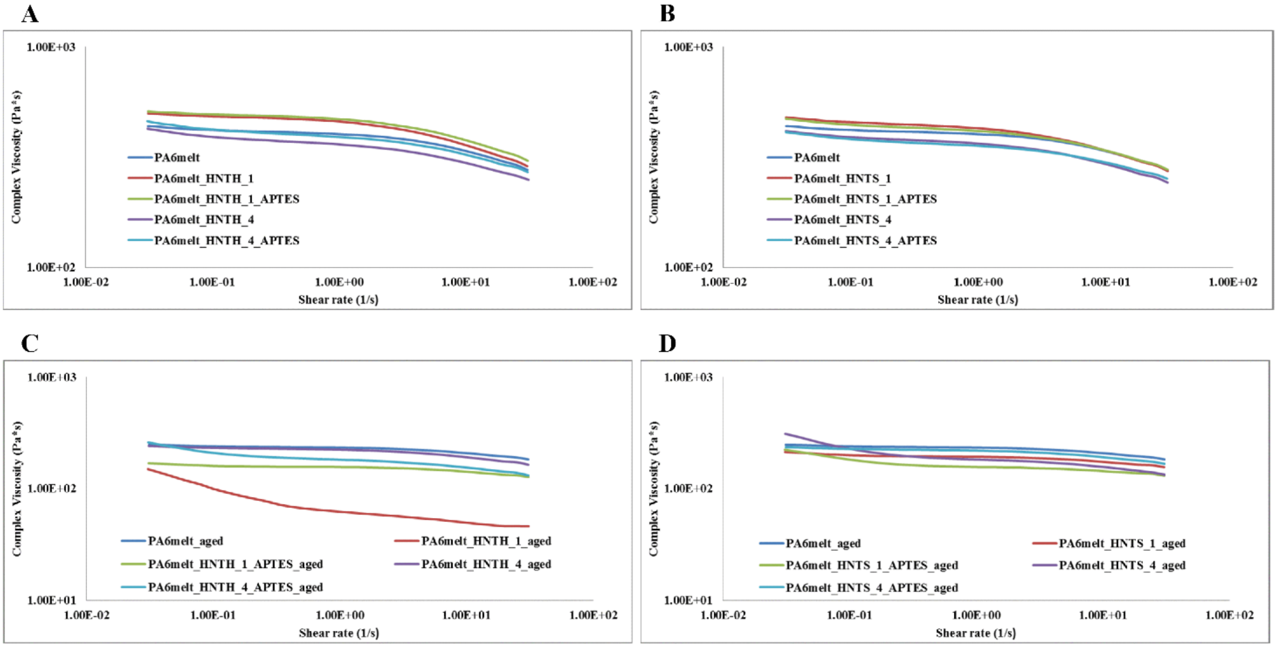

Figure S4. Rheological curves of **A**) PA6$_{melt}$_HNT$_{H}$, **B**) PA6$_{melt}$_HNT$_{S}$, **C**) PA6$_{melt}$_HNT$_{H}$_aged and **D**) PA6$_{melt}$_HNT$_{S}$_aged samples.

Table S4. Differential scanning calorimetry data of PA6_HNT nanocomposites collected during cooling and second heating thermal steps after the hydrothermal ageing test.

Sample	cooling		2nd heating		
	T_c (J/g)	ΔH_c (J/g)	T_m (J/g)	ΔH_f (J/g)	X_c (%)
PA6$_{in\ situ}$	189.3	64.6	222.1	−61.7	25.7
PA6$_{in\ situ}$_HNT$_{H}$_1	189.0	70.2	221.3	−61.6	25.7
PA6$_{in\ situ}$_HNT$_{S}$_1	187.4	52.8	224.3	−47.7	19.9
PA6$_{in\ situ}$_HNT$_{H}$_4	184.6	56.7	220.4	−47.3	19.7
PA6$_{in\ situ}$_HNT$_{S}$_4	185.7	63.9	221.9	−59.5	24.8
PA6$_{in\ situ}$_HNT$_{H}$_1_APTES	188.7	76.5	221.1	−48.0	28.0
PA6$_{in\ situ}$_HNT$_{S}$_1_APTES	188.5	70.5	222.7	−42.9	17.9
PA6$_{in\ situ}$_HNT$_{H}$_4_APTES	184.9	62.7	220.7	−31.9	13.3
PA6$_{in\ situ}$_HNT$_{S}$_4_APTES	184.7	66.8	221.7	−35.7	14.9
PA6$_{melt}$	190.5	62.9	222.8	−61.8	25.8
PA6$_{melt}$_HNT$_{H}$_1	192.5	71.5	221.0	−51.1	21.3
PA6$_{melt}$_HNT$_{S}$_1	191.6	69.9	221.5	−61.8	25.8
PA6$_{melt}$_HNT$_{H}$_4	190.2	68.1	221.4	−40.1	16.7
PA6$_{melt}$_HNT$_{S}$_4	190.7	65.0	222.0	−55.3	23.0
PA6$_{melt}$_HNT$_{H}$_1_APTES	191.4	71.6	221.2	−59.8	24.9
PA6$_{melt}$_HNT$_{S}$_1_APTES	192.7	80.4	220.8	−70.2	29.3
PA6$_{melt}$_HNT$_{H}$_4_APTES	187.9	75.4	221.2	−50.6	21.1
PA6$_{melt}$_HNT$_{S}$_4_APTES	188.8	67.0	222.8	−48.7	20.3

© 2020 by the authors. Submitted for possible open access publication under the terms and conditions of the Creative Commons Attribution (CC BY) license (http://creativecommons.org/licenses/by/4.0/).
